# A Genome-wide Association Study Provides Evidence of Sex-specific Involvement of Chr1p35.1 (*ZSCAN20-TLR12P*) and Chr8p23.1 (*HMGB1P46*) With Diabetic Neuropathic Pain

**DOI:** 10.1016/j.ebiom.2015.08.001

**Published:** 2015-08-04

**Authors:** Weihua Meng, Harshal A. Deshmukh, Louise A. Donnelly, Wellcome Trust Case Control Consortium 2 (WTCCC2), Surrogate markers for Micro- and Macro-vascular hard endpoints for Innovative diabetes Tools (SUMMIT) study group, Nicola Torrance, Helen M. Colhoun, Colin N.A. Palmer, Blair H. Smith

**Affiliations:** a Division of Population Health Sciences, Medical Research Institute, Ninewells Hospital and School of Medicine, University of Dundee, Dundee DD2 4BF, UK; b Centre for Pharmacogenetics and Pharmacogenomics, Medical Research Institute, Ninewells Hospital and School of Medicine, University of Dundee, Dundee DD1 9SY, UK

**Keywords:** Neuropathic pain, GWAS, Heritability, Sex-specific

## Abstract

Neuropathic pain is defined as pain arising as a direct consequence of a lesion or a disease affecting the somatosensory system and it affects around 1 in 4 diabetic patients in the UK. The purpose of this genome-wide association study (GWAS) was to identify genetic contributors to this disorder. Cases of neuropathic pain were defined as diabetic patients with a multiple prescription history of at least one of five drugs specifically indicated for the treatment of neuropathic pain. Controls were diabetic individuals who were not prescribed any of these drugs, nor amitriptyline, carbamazepine, or nortriptyline. Overall, 961 diabetic neuropathic pain cases and 3260 diabetic controls in the Genetics of Diabetes Audit and Research Tayside (GoDARTS) cohort were identified. We found a cluster in the Chr1p35.1 (*ZSCAN20-TLR12P*) with a lowest *P* value of 2.74 × 10^− 7^ at rs71647933 in females and a cluster in the Chr8p23.1, next to *HMGB1P46* with a lowest *P* value of 8.02 × 10^− 7^ at rs6986153 in males. Sex-specific narrow sense heritability was higher in males (30.0%) than in females (14.7%). This GWAS on diabetic neuropathic pain provides evidence for the sex-specific involvement of Chr1p35.1 (*ZSCAN20-TLR12P*) and Chr8p23.1 (*HMGB1P46*) with the disorder, indicating the need for further research.

## Highlights

• The case definition of diabetic neuropathic pain in this study is matched with those used in epidemiological studies.
• We confirmed that diabetic neuropathic pain is a heritable trait.
• We provided new genetic evidence of sex-specific involvement of two chromosome loci with diabetic neuropathic pain.

Using a pragmatic case definition, we identified two new genetic areas that may be involved in diabetic neuropathic pain, a common and debilitating complication of diabetes. One of these is more significant in males, the other in females. Furthermore, we calculated that the contribution of genes to developing neuropathic pain in diabetic men is about twice that in diabetic women. These findings help to explain why some people are more vulnerable to this complication, and help to elucidate the biological mechanisms of neuropathic pain. They will inform personalised (gender-specific) approaches to treatment, and the development of new drugs.

## 1 Introduction

Neuropathic pain is defined by the International Association for the Study of Pain as pain arising as a direct consequence of a lesion or a disease affecting the somatosensory system (Jensen et al., 2011). The prevalence of neuropathic pain is estimated to be around 7% in a general population while in a diabetic population around 1 in 4 patients will suffer from this disorder (van Hecke et al., 2014, Abbott et al., 2011). The current treatment of neuropathic pain is far from satisfactory, with fewer than 30% of patients achieving satisfactory relief of diabetic neuropathic pain (Barrett et al., 2007). Compared to people without pain and patients with non-neuropathic pain, diabetic neuropathic pain has a significant negative effect on patients' quality of life (Davies et al., 2006). In addition, the disorder represents a significant economic burden to healthcare systems (Tarride et al., 2006, Dworkin et al., 2010).

Cross-sectional epidemiological studies have identified multiple risk factors for neuropathic pain. These include older age, female gender, manual occupation, lower educational attainment, and living in a rural area or in poor accommodation (Smith et al., 2007, Torrance et al., 2006). These risk factors are difficult to modify and are not suitable for clinical intervention, though they are still of academic and political interest. Specifically for diabetic neuropathic pain, modifiable risk factors including smoking, hypertension, obesity, hypercholesterolemia and duration of diabetes have been identified (Jensen et al., 2006, Tesfaye et al., 2005). Unfortunately, there are no published clinical trials that suggest a reduction in the incidence or severity of neuropathic pain through addressing these modifiable risk factors. Further effort is required in this area. Studies have found that, although glycaemic control can reduce the incidence of diabetic neuropathy, there is limited impact in decreasing the incidence of accompanying neuropathic pain, even with long-term excellent glycaemic control (Callaghan et al., 2012, Marti et al., 2006). Epidemiological studies, such as genetic association studies, can identify independent risk factors which are clinically important, and offer these risk factors as covariates for basic research studies, or as new factors to address clinically.

Diabetic neuropathic pain is considered as a complex trait which is affected by both environmental risk factors and genetic risk factors. Unlike well documented environmental risk factors, the understanding of the genetic contributors to neuropathic pain is rather poor, though evidence from animal models and human studies have both confirmed that it is a heritable trait (Devor et al., 2005, Meng et al., 2015). Studies on animal models have proposed candidate genes for neuropathic pain such as *P2X7*, *P2X4*, *TLR4*, and *CACNG2* (Chessell et al., 2005, Trang et al., 2009, Nissenbaum et al., 2010, Wang et al., 2013). The first genome-wide association study (GWAS) on diabetic neuropathic pain in humans reported that *GFRA2* might be associated with a subgroup of this disorder (Meng et al., 2015). All these candidate genes need further replications to validate their biological roles.

We conducted a population-based GWAS of diabetic neuropathic pain in which our case definition was matched with previous population-based observational studies of diabetic neuropathic pain (Hall et al., 2013, Dieleman et al., 2008), seeking candidate genes that might not have been identified using the previous, more exclusive case definition (Meng et al., 2015).

## 2 Methods

### 2.1 Resources

Genetic resources: The Genetics of Diabetes Audit and Research Tayside (GoDARTS) project recruits diabetic patients and non-diabetic matched controls in Tayside, Scotland to identify genetic contributors relating to the susceptibility of diabetes, the complications of diabetes, the response to diabetes treatment and the prognosis of diabetes. (http://diabetesgenetics.dundee.ac.uk/). So far, the project has recruited 9439 diabetic patients who have provided their DNA samples along with written consent to use their clinical data and biological samples for research. Among these 9434 diabetic individuals, 3673 were genotyped by Affymetrix SNP6.0 chips supported by the Wellcome Trust Case Control Consortium 2 (WTCCC2) project (http://www.wtccc.org.uk/ccc2/) and 3254 were genotyped by Illumina OmniExpress chips supported by the Surrogate markers for Micro- and Macro-vascular hard endpoints for Innovative diabetes Tools (SUMMIT) project (http://www.imi-summit.eu/). The GoDARTS study was approved by Tayside Committee on Medical Research Ethics (REC reference 053-04).

E-health resources: Since 1993, every person registered with the National Health Service (NHS) in Scotland has been assigned a unique Community Health Index (CHI) number. This number appears in the records of all personal medical activities within the NHS framework which paves the way for anonymous data linkage. The GoDARTS project includes consent from participants for the genetic data to be anonymously linked with datasets sourced from participants' NHS medical histories, including prescribing data, blood test results, radiology examination results, hospital admissions, and outpatient appointments. The current prescription history dataset used in this study covers from Jan, 1993 to Dec, 2013.

## 3 Definitions of Cases and Controls of Neuropathic Pain

In this study, we defined a neuropathic pain case as a type 2 diabetic patient who has a history of multiple usages (minimum twice) of at least one of the following five medicines which are recommended and effective in diabetic peripheral neuropathy and prescribed uncommonly for other disorders: duloxetine, gabapentin, pregabalin, capsaicin cream (or patch) and lidocaine patch (Attal et al., 2010, National Institute for Health and Care Excellence NICE (UK), 2013; Finnerup et al., 2010).

A control was defined as a type 2 diabetic patient who has not been prescribed any of these five drugs before. Individuals who had a prescription history of amitriptyline, carbamazepine, or nortriptyline were not included as controls because these drugs are often used for the treatment of other medical conditions, as well as neuropathic pain. In other words, diabetic individuals using these drugs could be correctly classified as neuropathic pain cases or wrongly classified if these drugs were used for treating other disorders such as depression or epilepsy. It is not possible to differentiate these two situations with certainty based on the available clinical information. To avoid the potential for incorrect phenotyping, those individuals were also removed from the control group.

We excluded individuals with a history of only one single prescription for any of these five drugs from both cases and controls.

### 3.1 Genotyping and Quality Control

The quality control steps of the genotype data were applied based on the standard methods that were used for the WTCCC2 studies (GoDARTS & UKPDS Diabetes Pharmacogenetics Study Group et al., 2011), and the SUMMIT studies (Fagerholm et al., 2012).

### 3.2 Statistical Analysis

**Fig. 1:**
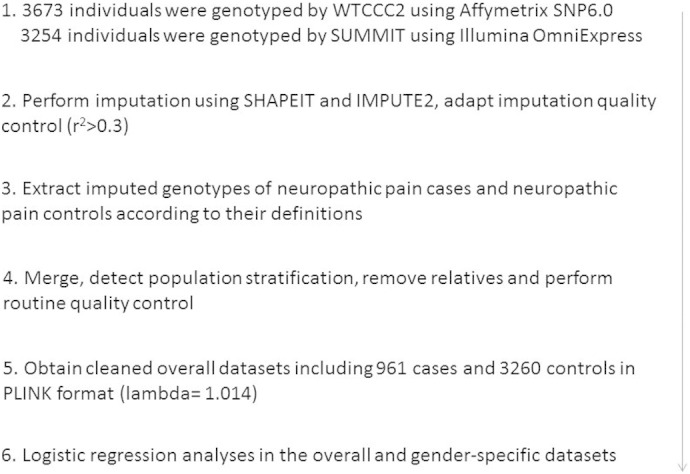
Workflow of the GWAS on neuropathic pain.

Non-genotyped single nucleotide polymorphisms (SNPs) in the Affymetrix SNP6.0 chips and Illumina OmniExpress chips were imputed by SHAPEIT and IMPUTE2 based on shared reference files from the 1000 genome phase I datasets (Delaneau et al., 2011, Howie et al., 2009). An r^2^ score was used to assess the accuracy of an imputed genotype. It is suggested to adopt an r^2^ > 0.3 to remove imputed SNPs with poor quality. PLINK was the main GWAS software for genetic data manipulation and standardised quality control steps were frequently performed during analyses (For example, SNPs with over 10% genotyping missing were excluded, SNPs with minor allele frequency less than 1% were removed, SNPs which failed Hardy-Weinberg tests (*P* < 0.000001) were removed, and individuals with more than 10% genotype data missing were not included) (Purcell et al., 2007). SNPs on the sex chromosomes and mitochondrial SNPs were not included in the analyses since we do not have these data. The detection of individuals with different ancestry was done by the multidimensional scaling method implanted in PLINK. A lambda value generated by this method indicates the level of population stratification. The lambda value should be very close to 1 in a homogeneous population with little ancestry mixture. Related samples were identified by calculating pi-hat values greater than 0.125 in PLINK. Logistic regression analyses were applied to generate *P* values for SNP association tests. A *P* value of less than 10^− 6^ was considered to be a suggestive association, worth further exploration. SNP functional annotation was searched by SNPnexus and Manhattan plots were generated by HaploView (Barrett et al., 2005, Dayem Ullah et al., 2013). Regional visualisation was achieved by LocusZoom (Pruim et al., 2010). The Q–Q plot of *P* values, a tool to assess whether there are confounders and the impact of these potential confounders (different genotyping machines, different genotyping chips, different DNA extraction methods, etc) between cases and controls, was visualised by SNPEVG (Wang et al., 2012). The whole workflow is summarised in Fig. 1. Narrow-sense heritabilities of the overall dataset and sex-specific dataset were performed by restricted maximum likelihood analysis using the recognized approach to genome-wide complex trait analysis (GCTA) (Lee et al., 2011). Narrow-sense heritability represents the ratio of total phenotypic variance which is caused by additive genetic effects of individual SNPs (Lee et al., 2011). Comparisons of means of age and BMI between cases and controls were performed using independent t test in SPSS 21 (IBM Corp, New York, USA). The gender difference was evaluated using chi-square (2 × 2 tables).

## 4 Results

We identified 1043 diabetic patients who had a prescription record of minimum twice usage of at least one of the five relevant neuropathic pain drugs (Duloxetine, Gabapentin, Pregabalin, Capsaicin cream (or patch) and Lidocaine patch, see Methods section for details) among the genotyped diabetic population of the GoDARTS project, representing 15.06% of the cohort. In addition, we found 3759 diabetic individuals who were identifiable as controls, as they had not been prescribed any of these five drugs, nor other drugs that can be used (non-exclusively) to treat neuropathic pain (amitriptyline, carbamazepine, or nortriptyline). After removing ethnically outlying samples, genetically related samples, type 1 diabetic samples and those who had had a single prescription of neuropathic pain drugs, the final cohort for analysis comprised 961 neuropathic pain cases (male = 470, female = 491) and 3260 controls (male = 2021, female = 1239). We then derived data summarising the age and body mass index (BMI) for the overall dataset, male only dataset and female only dataset (Table 1). In the overall dataset, the average age (mean ± standard deviation, years) and BMI (mean ± standard deviation, kg/m^2^) in cases were 72.60 ± 10.54, and 27.79 ± 6.01, respectively. The average age and BMI in controls were 75.51 ± 10.79, and 26.91 ± 5.51, respectively. There were statistically significant differences in age and BMI between cases and controls as well as in gender (*P* < 0.01). In the male only dataset, the average age and BMI in cases were 72.71 ± 9.96, and 27.06 ± 5.01, respectively. The average age and BMI in controls were 74.82 ± 10.69, and 26.83 ± 4.94, respectively. There was no statistical difference in BMI between cases and controls, but the difference in age was statistically significant (*P* < 0.01). In the female only dataset, the average age and BMI in cases were 72.48 ± 11.08, and 28.49 ± 6.56, respectively. The average age and BMI in controls were 76.63 ± 10.90, and 27.06 ± 6.33, respectively. The differences in age and BMI between cases and controls were statistically significant (*P* < 0.01).

**Fig. 2:**
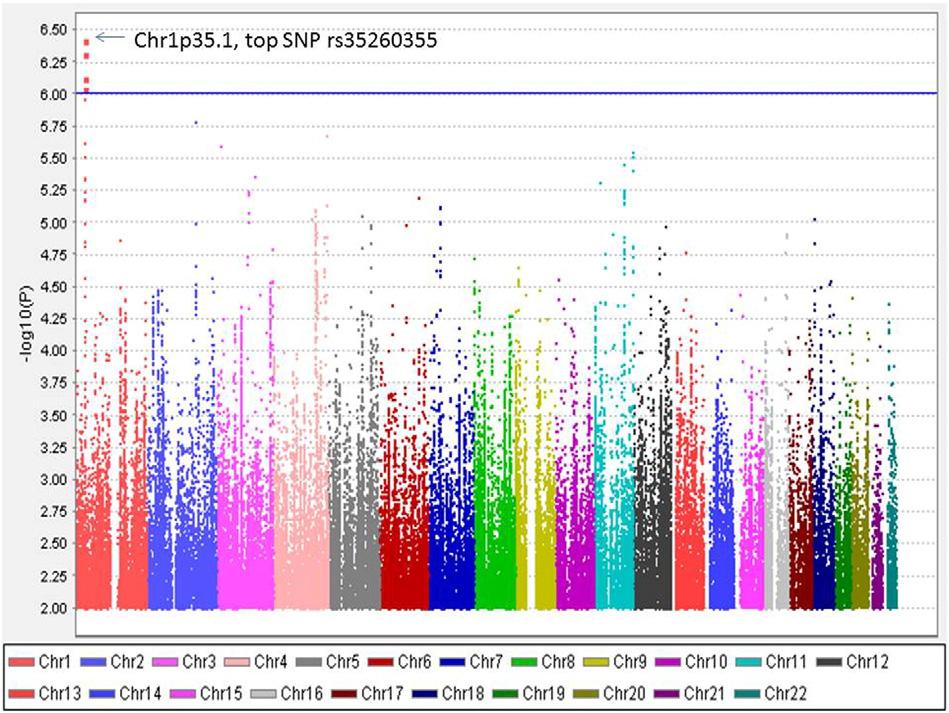
Manhattan plot of the GWAS on neuropathic pain in the overall dataset. X axis represents 22 autosomes. Y axis means the –log10 of *P* values. The blue line is the cut-off *P* value of 10^− 6^. Cases and controls included 961 and 3260 samples, respectively. (Only SNPs whose *P* < 0.01 were used to make the plot).

**Fig. 3:**
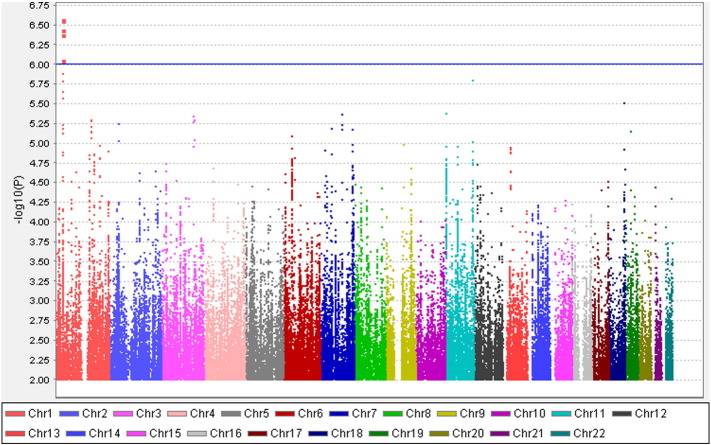
Manhattan plot of the GWAS on neuropathic pain in the female only dataset. X axis represents 22 autosomes. Y axis means the –log10 of *P* values. The blue line is the cut-off *P* value of 10^− 6^. Cases and controls included 491 and 1239 individuals, respectively. (Only SNPs whose *P* < 0.01 were used to make the plot).

**Fig. 4:**
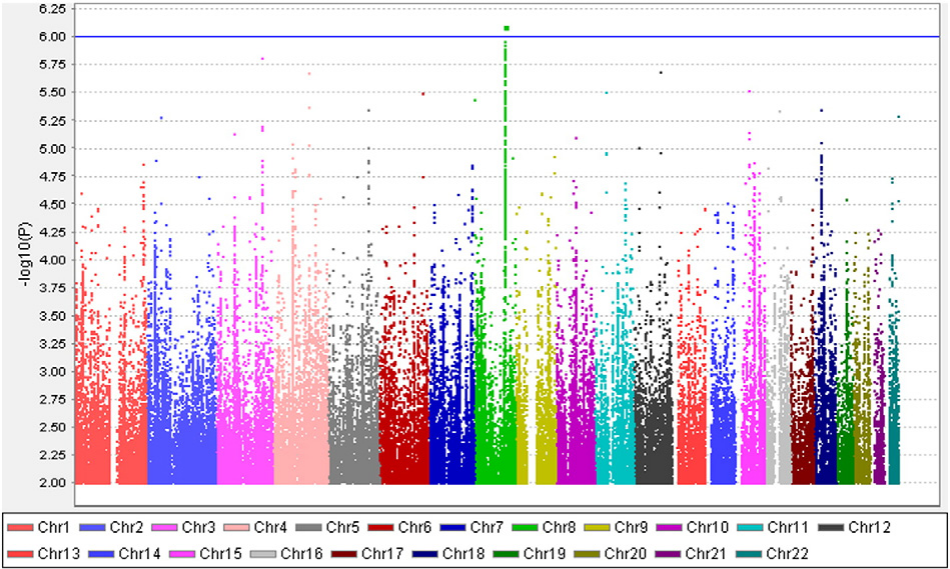
Manhattan plot of the GWAS on neuropathic pain in the male only dataset. X axis represents 22 autosomes. Y axis means the –log10 of *P* values. The blue line is the cut-off *P* value of 10^− 6^. Cases and controls included 470 and 2021 individuals, respectively. (onLy SNPs whose P < 0.01 were used to make the plot).

**Fig. 5:**
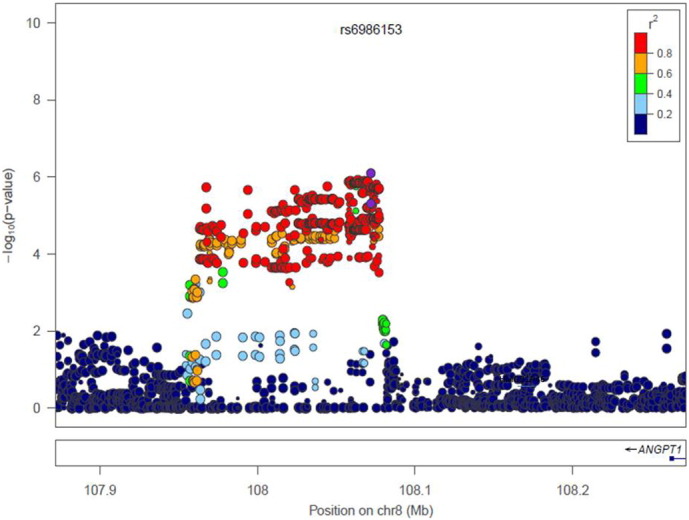
Regional plot of Chr1p35.1 in females. r^2^ represents the linkage disequilibrium among SNPs.

**Fig. 6:**
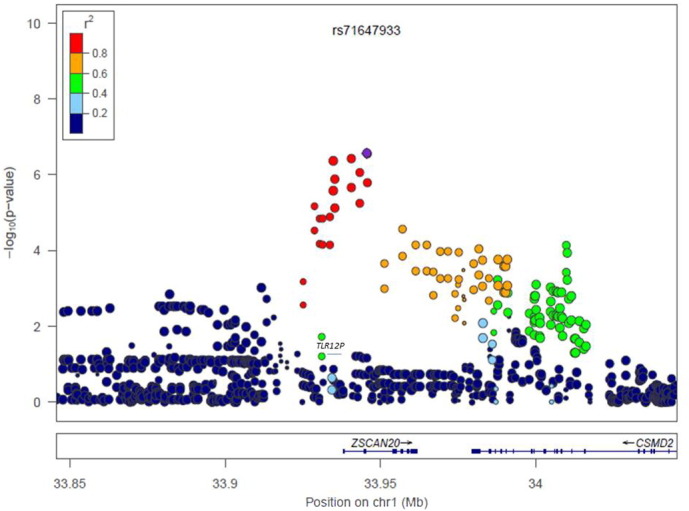
Regional plot of Chr8p23.1 in males. r^2^ represents the linkage disequilibrium among SNPs.

Altogether 6,906,962 genotyped and imputed SNPs survived for analysis, after standardised quality control of genotyping and imputation (r^2^ > 0.3). Since the lambda value (indicating the level of population stratification) was 1.014 for the cleaned overall dataset, no extra adjustment was adopted based on population stratification. Using logistic regression testing, with age, sex, and BMI as covariates for the overall dataset, there was a peak showing in chromosome 1 on the Manhattan plot (Fig. 2). The associated Q–Q plot is shown in Supplementary File 1. Although none of the SNPs reached formal genome-wide significance (5 × 10^− 8^), the cluster in Chromosome 1p35.1 (Chr1p35.1), spanning *ZSCAN20-TLR12P* area, still indicated possible associations. The most significant SNP in this region was rs35260355 in the *ZSCAN20* with a lowest *P* value of 3.84 × 10^− 7^ and an odds ratio (OR) of 1.66 (95% confidence interval: 1.37–2.02). Similar logistic regression in the female only dataset found that the peak in the Chr1p35.1 still existed and the top SNP rs71647933 in the *ZSCAN20* achieved a lower *P* value of 2.74 × 10^− 7^ with an OR of 2.31 (95% confidence interval: 1.68–3.17) (Fig. 3). In the male only dataset, the SNP cluster in the Chr1p35.1 disappeared while a new peak showed in the Chr8p23.1, next to *HMGB1P46* and the *P* value of the top SNP rs6986153 was 8.02 × 10^− 7^ with an OR of 1.67 (95% confidence interval: 1.34–2.08) (Fig. 4). Table 2 summarises all the significant SNPs found in the regions in the three datasets. Fig. 5, Fig. 6 show the regional plots of the identified loci in the female only dataset and the male only dataset, respectively. It was estimated that the narrow-sense heritability of neuropathic pain was 14.7% in the overall dataset, but 30.0% among males, compared with 14.7% among females.

## 5 Discussion

Utilising a genetic dataset and e-health linkage dataset, we performed a GWAS on diabetic neuropathic pain using case and control definitions matched with previous population-based epidemiological studies and the results suggested two loci that may be involved with painful diabetic neuropathy.

Standard protocols of the assessment of neuropathic pain have been widely agreed for specialist settings and primary care (Haanpää et al., 2011, Jones and Backonja, 2013). However, there is no common approach or consensus reached by clinicians or researchers to define neuropathic pain in population-based settings or in general cohorts. As GoDARTS participants were recruited through community-based clinics and general hospitals, there is no formal record of neuropathic pain status made by specialists. We acknowledge that expert clinical examination would have increased the robustness of the case definition in this cohort. However, without clinical examination evidence, it is reasonable to use an alternative, acceptable definition to represent neuropathic pain cases. We adopted a pragmatic approach to define cases using a multiple prescription history of the five main drugs used exclusively or mainly to treat neuropathic pain (rather than other disorders) in a diabetic population. A combination of diagnostic codes for type 2 diabetes and prescription of neuropathic pain drugs has been used in previous epidemiological studies to identify patients with painful diabetic neuropathy (Hall et al., 2013, Dieleman et al., 2008). Members of our population-based cohort were already identified as having type 2 diabetes, and so our method of identifying neuropathic pain makes this study reasonably consistent with these previous studies. While amitriptyline, carbamazepine, and nortriptyline are also frequently used in neuropathic pain, we considered that these are relatively likely to be used for indications other than neuropathic pain and we did not include individuals who had been prescribed these drugs as either cases or controls. To have a more homogeneous population, we removed individuals with only a single prescription of the five neuropathic pain drugs from both cases and controls. It has previously been highlighted that patients in primary care with neuropathic pain are often not prescribed any of the specific medications for its treatment (Hall et al., 2008, Torrance et al., 2007, Torrance et al., 2013). As there is no pain status recorded in the GoDARTS, no direct assessment of the presence of (neuropathic) pain can be made among cases or controls. Furthermore, we did not assess whether cases or controls had received any other prescriptions for pain, such as opioid medications, and it is possible that some with neuropathic pain were treated with drugs that are not specifically indicated for this. Therefore the definition in our study is possible to have classified some who have neuropathic pain as controls but few controls as cases. The subsequent *P* values and ORs may be underestimated, though we cannot measure the extent of this.

The most significant SNP cluster in the overall dataset was found in Chr1p35.1 with a lowest *P* value of 3.84 × 10^− 7^ at rs35260355, spanning *ZSCAN20-TLR12P* area. The function of *ZSCAN20* (zinc finger and SCAN domain containing 20) gene is not known yet and it has not been noted to be associated with any disorders. One of the proteins it codes contain typical C2H2 zinc finger domain, which enables zinc finger protein to bind other molecules such as RNA and DNA and affect transcription and translation (Krishna et al., 2003). There have been attempts to use zinc finger proteins to treat neuropathic pain since the receptor specific transcription factors of zinc-finger proteins have been developed to target gene repression in cell line models and in vitro (Tan et al., 2005). It is worth noting that the top SNP from the female only dataset rs71647933 is suggested to be a transcription factor binding site of the zinc interaction domain (SNPnexus). Toll-like receptors (TLRs) are a class of proteins which exist in various cell types in the central nervous system, including neuronal and non-neuronal cells (Liu et al., 2012). TLRs share structural and functional similarities. Specifically, the deletion or inhibition of TLR2 and TLR4 in animal models will impair nerve injury-induced neuropathic pain (Kim et al., 2007, Tanga et al., 2005). When using a TLR4 antagonist to treat both wild type mice and *TLR4* knockout mice suffering neuropathic pain, pain relief can be achieved in the wild type mice but not in the *TLR4* knockout mice (Bettoni et al., 2008). *TLR12P* is a unitary pseudogene with a transcript but there is no protein product of this gene in the human. The function of its homolog in mice is unclear although it is suggested it may be involved in the immune system against pathogens (Koblansky et al., 2012). There is emerging evidence showing that *TLRs* are involved in the control of (neuropathic) pain while the mechanisms are still far from being elucidated (Liu et al., 2012). In the females only dataset (1730 individuals), the *P* value of the SNPs in the cluster were lower than in the overall dataset, indicating that the male samples were not contributing so much to the associations in this cluster, and that the identified *ZSCAN20-TLR12P* locus has a gender specific influence on diabetic neuropathic pain. This is consistent with the findings of other *TLR* genes. Studies have found that variants in *TLR* genes are gender-specifically linked with multiple situations (Roberts et al., 2012). The mechanism of sex-specific phenomena is not clear and the evidence for hormone involvement is insufficient and controversial (Roberts et al., 2012, Berghöfer et al., 2006).

We also identified a peak in the Chr8p23.1 next to *HMGB1P46* when analysing the male only dataset, and the *P* value of the top SNP rs6986153 was 8.02 × 10^− 7^ with an OR of 1.67. *HMGB1P46* is a pseudogene of high mobility group box-1 (*HMGB1*). It is suggested that the induction of high mobility group box-1 in the dorsal root ganglion can contribute to pain hypersensitivity after peripheral nerve injury (Shibasaki et al., 2010). In addition, Feldman et al found that the persistent endogenous release of HMGB1 by sensory neurons contributes to tactile hyperalgesia in a neuropathic pain rat model (Feldman et al., 2012). The synthesis and release of HMGB1 from spinal neurons due to nerve injury facilitates the activity of both microglia and neurons which leads to symptoms of neuropathic pain (Nakamura et al., 2013). It is interesting to know that HMGB1 signalling and TLR pathways, to some extent, are overlapping together (Yu et al., 2006, Velegraki et al., 2012). There is evidence that pseudogenes are involved in the biological process. For example, the low level of high mobility group A1 (*HMGA1*) was also associated with a high level of *HMGA1* pseudogene (HMGA1-p) mRNA (Chiefari et al., 2010). It was observed that knockdown of HMGA1-p RNA in the cells of diabetic patients led to partially restored HMGA1 mRNA levels which suggested a competing relationship between the two types of transcripts. It is therefore hypothesised that a competing relationship might also exist between HMGB1 and its pseudogenes.

There were no SNPs found with a *P* value of less than 5 × 10^− 8^ in the overall dataset, male only or female only datasets. Although a *P* value of 5 × 10^− 8^ is generally adopted as the cut-off *P* value for GWAS significance, it has been suggested that this might be too stringent and risks missing important associations (i.e. false negatives) (Do et al., 2014). Using a lower threshold raises the chance of detecting associated SNPs, but also of detecting spurious associations (false positives), and we need to beware of that in interpreting this study. The narrow-sense heritability (variance explained by SNPs, excluding genetic variation due to dominance, epistasis, and environment) of diabetic neuropathic pain in the overall dataset was estimated to be 14.7%, which is similar to that found in our previous analysis (Meng et al., 2015). However when calculated by gender, we found males had a higher heritability (30.0%) than females (14.7%). Sex-specific heritability has been observed in other traits (Weiss et al., 2005). The reasons behind the different gender-specific heritabilities are unknown although it may result from parent-of-origin effects, interaction with sex chromosomes and the sex-specific hormonal environment. It is worth considering sex-specific genetic effects in future association studies of neuropathic pain. There are some reports indicating that genetic effects are different between genders in determining pain. Experiments in mice found that the Mc1r gene mediates kappa-opioid analgesia in female mice only. Correspondingly in a human study, females with two variant *MC1R* alleles showed greater analgesic responses from the kappa-opioid, pentazocine, than males and females who did not have the variant alleles (Mogil et al., 2003). In addition, polymorphisms in the *OPRM1* gene have been reported to be associated with pressure-related pain sensitivity in men but not in women (Fillingim et al., 2005). Sato et al found that there were significant associations between the opioid receptor genes (*OPRM1*, *OPRD1* and opioid *OPRK1*) and experimental pain sensitivity (Sato et al., 2013). Our results showing genetic differences associated with neuropathic pain between genders are consistent with these findings, though the biological mechanisms remain unclear and highlight the need for further research in this area. The heritability of neuropathic pain has been calculated as around 30% in rat models (Devor et al., 2005), similar to that measured here among men, though twice that found among women..

Using an additive model integrated in the CaTS, we had 80% power for the overall dataset (961 cases and 3260 controls), assuming a minor disease allele frequency of 0.20, a genotypic relative risk for this variant of 1.31, a prevalence of neuropathic pain in the diabetic population of 0.25, and the significance level is 10^− 6^ (Skol et al., 2006). In our previous analysis, our case definition also included evidence of neuropathy, based on recorded results of monofilament testing (Meng et al., 2015). As we did not consider the results of monofilament testing in this study, our case definition was more inclusive and therefore less specific. Although there are power benefits of including more cases, there is also a possibility that neuropathic pain with and without neuropathy evidence might have separate genetic risk markers, as well as shared genetic mechanisms. No studies have been reported examining whether there is any genetic difference between neuropathic pain with and without neuropathy evidence. The peaks we have identified in this paper could reflect some ‘general’ genetic mechanisms of neuropathic pain while the different peaks identified in our previous GWAS may be specifically associated with neuropathic pain with neuropathy evidence (Meng et al., 2015). In other disorders, a phenotype and its subtypes have been shown to have both shared and different genetic risks (Kessler et al., 2013). Similarly, we did not remove those who were prescribed strong opioid drugs from the control group since opioid drugs are neither indicated first- or second-line treatments, nor commonly used to treat diabetic neuropathic pain (Torrance et al., 2013). A good phenotype, endophenotype and subgroup definition should aim to reflect the underlying genetic mechanisms.

There are some recent GWAS published in the field of pain research. A locus between *CCT5* and *FAM173B* located at Chr5p15.2 has been proposed to be associated with chronic widespread pain (Peters et al., 2013). *TAOK3* was suggested to be associated with morphine requirement and postoperative pain in a retrospective paediatric day surgery population (Cook-Sather et al., 2014). Rs11127292 in the *MYT1L* was found to be associated to fibromyalgia with low comorbidities (Docampo et al., 2014). Another GWAS study suggested rs2952768 in the Chr2q33.3 was involved with analgesic requirements in humans (Nishizawa et al., 2014). These GWAS have shed light on the elucidation of the genetic pathways for pain while further research is needed, including replication studies, functional studies, and agreement on feasible, valid and reproducible phenotype ascertainment.

The limitations of our study include that the *P* values of tops SNPs are only close to GWAS significance but yet reached; no replication study to confirm the results; though the case definition is matched with those used epidemiological studies, we might misclassify some cases who have neuropathic pain but not prescribed medications into controls; we might also misclassify an individual into a control group who uses opioid to treat neuropathic pain.

We have provided genetic evidence that SNPs in Chr1p35.1 (*ZSCAN20-TLR12P*) and Chr8p23.1 (*HMGB1P46*) may be involved with neuropathic pain in diabetes. Sex-specific associations are also suggested. Our findings should be treated with caution and, while we have also presented their consistency with known biological factors, they can only guide the nature of future research, which will be based on the findings reported in this paper. Any replication of our findings will help to confirm hypothesised pathways involved in the genetic mechanisms of neuropathic pain and provoke research on new potential drug targets for the treatment of pain.

The following is the supplementary data related to this article.

**Supplementary figure:**
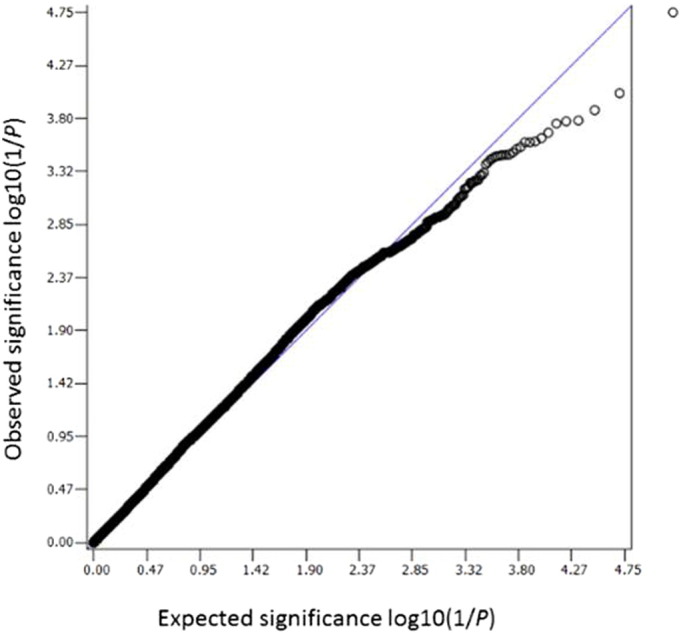
Q–Q plot expected and observed log10(1/P) values. Only SNPs with *P* values less than 0.01 were used and *P* values were multiplied to 100 before plotting.

Supplementary data to this article can be found online at http://dx.doi.org/10.1016/j.ebiom.2015.08.001.

## Competing Interests

The authors declare that they have no competing interests.

## Author Contributions

WM analysed the data and prepared the manuscript. HD contributed to the imputation dataset. YL contributed to e-health linkage dataset. HC read the paper and provided suggestions to the discussion. NT and CP reviewed the paper and made a contribution to the discussion. BS contributed to the design of the study, and contributed significantly to the manuscript.

## Role of the Funding Sources

Funding sources did not have any involvement in the study design; the collection, analysis and interpretation of data; writing of the report; or the decision to submit the article for publication.

## Figures and Tables

**Table 1:** Information on covariates between cases and controls.

		Cases	Controls	*P* value
Overall dataset	Age	72.60 ± 10.54	75.51 ± 10.79	< 0.01
BMI	27.79 ± 6.01	26.91 ± 5.51	< 0.01
Male:Female	470:491	2021:1239	< 0.01
Male only	Age	72.71 ± 9.96	74.82 ± 10.69	< 0.01
BMI	27.06 ± 5.01	26.83 ± 4.94	> 0.05
Female only	Age	72.48 ± 11.08	76.63 ± 10.90	< 0.01
BMI	28.49 ± 6.56	27.06 ± 6.33	< 0.01

Age and BMI (body mass index) are presented as mean ± standard deviation.

Age is defined as 2014 — birth year.

**Table 2:** Significant SNPs in the overall, female only and male only dataset.

Dataset	SNP	Chr	Position	Gene	Minor allele	Minor allele frequency in cases:controls	*P* value	OR (95% CI)
Overall	rs4652898	1	33940691	*ZSCAN20*	*C*	0.19:0.16	7.45 × 10^− 7^	1.63 (1.34–1.98)
rs2336244	1	33943390	*ZSCAN20*	*C*	0.18:0.15	9.07 × 10^− 7^	1.67 (1.36–2.05)
rs71647933	1	33945601	*ZSCAN20*	*G*	0.19:0.16	4.88 × 10^− 7^	1.65 (1.36–2.02)
rs35260355	1	33945831	*ZSCAN20*	*T*	0.19:0.16	3.84 × 10^− 7^	1.66 (1.37–2.02)
Female	rs10914731	1	33934824	Intergenic	*G*	0.21:0.16	4.25 × 10^− 7^	2.25 (1.64–3.09)
rs4652898	1	33940691	*ZSCAN20*	*C*	0.20:0.16	3.70 × 10^− 7^	2.29 (1.67–3.16)
rs2336244	1	33943390	*ZSCAN20*	*C*	0.19:0.15	9.00 × 10^− 7^	2.39 (1.69–3.38)
rs71647933	1	33945601	*ZSCAN20*	*G*	0.20:0.16	2.74 × 10^− 7^	2.31 (1.68–3.17)
rs35260355	1	33945831	*ZSCAN20*	*T*	0.20:0.16	2.81 × 10^− 7^	2.30 (1.68–3.17)
Male	rs6986153	8	108072044	Intergenic	*G*	0.27:0.19	8.02 × 10^− 7^	1.67 (1.34–2.08)

Chr, chromosome; SNP, single nucleotide polymorphisms; OR, odds ratio; 95% CI, 95% confidence interval.

*P* values and ORs were calculated using logistic regression test.
